# The UK Crop Microbiome Cryobank: a utility and model for supporting Phytobiomes research

**DOI:** 10.1186/s43170-023-00190-2

**Published:** 2023-11-26

**Authors:** Matthew J. Ryan, Tim H. Mauchline, Jacob G. Malone, Susan Jones, Catriona M. A. Thompson, J. Miguel Bonnin, Helen Stewart, Payton T. O. Yau, Rodrigo G. Taketani, Ian M. Clark, Nicola Holden

**Affiliations:** 1 CABI, Bakeham Lane, Egham, Surrey, TW20 9TY UK; 2 Rothamsted Research, West Common, Harpenden, Hertfordshire, AL5 2JQ UK; 3 John Innes Centre, Norwich Research Park, Colney Ln, Norwich, NR4 7UH UK; 4 School of Biological Sciences, University of East Anglia, Norwich, NR4 7TJ UK; 5 Information and Computational Sciences, The James Hutton Institute, Invergowrie, Dundee, DD2 5DA UK; 6 Scotland’s Rural College (SRUC), AB21 9YA Aberdeen, UK

**Keywords:** Rhizosphere, Microbiota, Biobank, Metagenome, Microbiome, Soil health

## Abstract

Plant microbiomes are the microbial communities essential to the functioning of the phytobiome—the system that consist of plants, their environment, and their associated communities of organisms. A healthy, functional phytobiome is critical to crop health, improved yields and quality food. However, crop microbiomes are relatively under-researched, and this is associated with a fundamental need to underpin phytobiome research through the provision of a supporting infrastructure. The UK Crop Microbiome Cryobank (UKCMC) project is developing a unique, integrated and open-access resource to enable the development of solutions to improve soil and crop health. Six economically important crops (Barley, Fava Bean, Oats, Oil Seed Rape, Sugar Beet and Wheat) are targeted, and the methods as well as data outputs will underpin research activity both in the UK and internationally. This manuscript describes the approaches being taken, from characterisation, cryopreservation and analysis of the crop microbiome through to potential applications. We believe that the model research framework proposed is transferable to different crop and soil systems, acting not only as a mechanism to conserve biodiversity, but as a potential facilitator of sustainable agriculture systems.

## Introduction

Culture collections have long supported microbiological research through the supply and conservation of microbes in axenic (pure) format. However, the rapid advancement of sequencing technology as a mechanism to characterise communities of microbes, hereafter the microbiota, has not been correlated with the development of supporting infrastructure. The key requirements for biobanking for microbiome research have been defined and include the development of standards, the need to deposit material and to supply cultures, samples and associated data for future research in both academia and industry (Ryan et al. 2021). By default, this also provides a mechanism to protect intellectual property as part of patent deposit protocols, and help researchers adhere to legislative and regulatory requirements including the Nagoya Protocol of the Convention of Biological Diversity (CBD). While soil biobanks, such as the Rothamsted Soil Archive (Clark and Hirsch 2008) exist, conservation of microbes within the soil is not the primary purpose of storing the samples.

Supporting microbiome research presents as a more challenging problem—how do we translate the methodology used by culture collections to complex samples that may contain many thousands of different species? Integral to the above is the need to preserve soil and plant samples, their microbiota and associated metadata. This needs a new approach to consider how samples are processed and analysed, how they are cultured and preserved and finally how the sequence datasets generated are made available to researchers and integrated within public repositories. Other than experimental datasets from individual experiments, there are currently few microbiome-focussed collections (Ryan et al. 2021) and there are none with a focus on the soil-crop microbiome. The UK Crop Microbiome Cryobank (UKCMC), a publicly funded initiative, is therefore unique and seeks to fill this gap through the establishment of a cryopreserved resource of characterized material from soil/root crop microbiomes. The UKCMC has a prioritized collection strategy, consisting of fungi, bacteria and rhizosphere microbiome samples. Details of our physically cryopreserved samples and their associated datasets can be accessed through AgMicroBiomeBase (https://agmicrobiomebase.org/). Methodologies, for collection and storage of intact microbial communities in environmental samples and extracts of total DNA, will be made available through this resource. We are using advanced cryopreservation technology to develop regimes which will enable us to sustainably maintain the resource in a genotypically and phenotypically stable state. All resources are being genomically characterised, allowing for assessments, from whole community taxonomies (bacterial and fungal amplicons), metagenomes, to individual isolate genomes. One benefit of the resource is the potential to find new biological-based products (whole organisms and natural products) which may facilitate public–private partnerships in the Agritech sector. This will be demonstrated to the user community, as proof of principle, through Plant Growth Promoting Rhizobacteria (PGPR) screening and synthetic community construction.

This short communication describes the approaches used in the project, its aims in supporting the microbiome research community, and how the model will be useful for application in other crop systems and the importance of biobanking per se*.*

## Samples and cultures

**Fig. 1:**
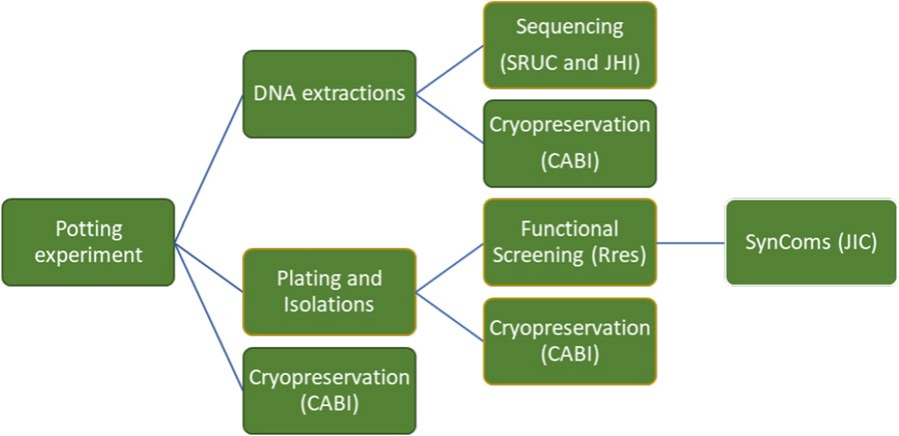
Project workflow, the success of the project workplan is dependent on sample processing and culturing, each partner is responsible for a specific work-package. *CABI* Centre for Agricultural Bioscience International, *JHI* James Hutton Institute, *JIC* John Innes Centre, *Rres* Rothamsted Research, *SRUC* Scotland’s Rural College

**Fig. 2:**
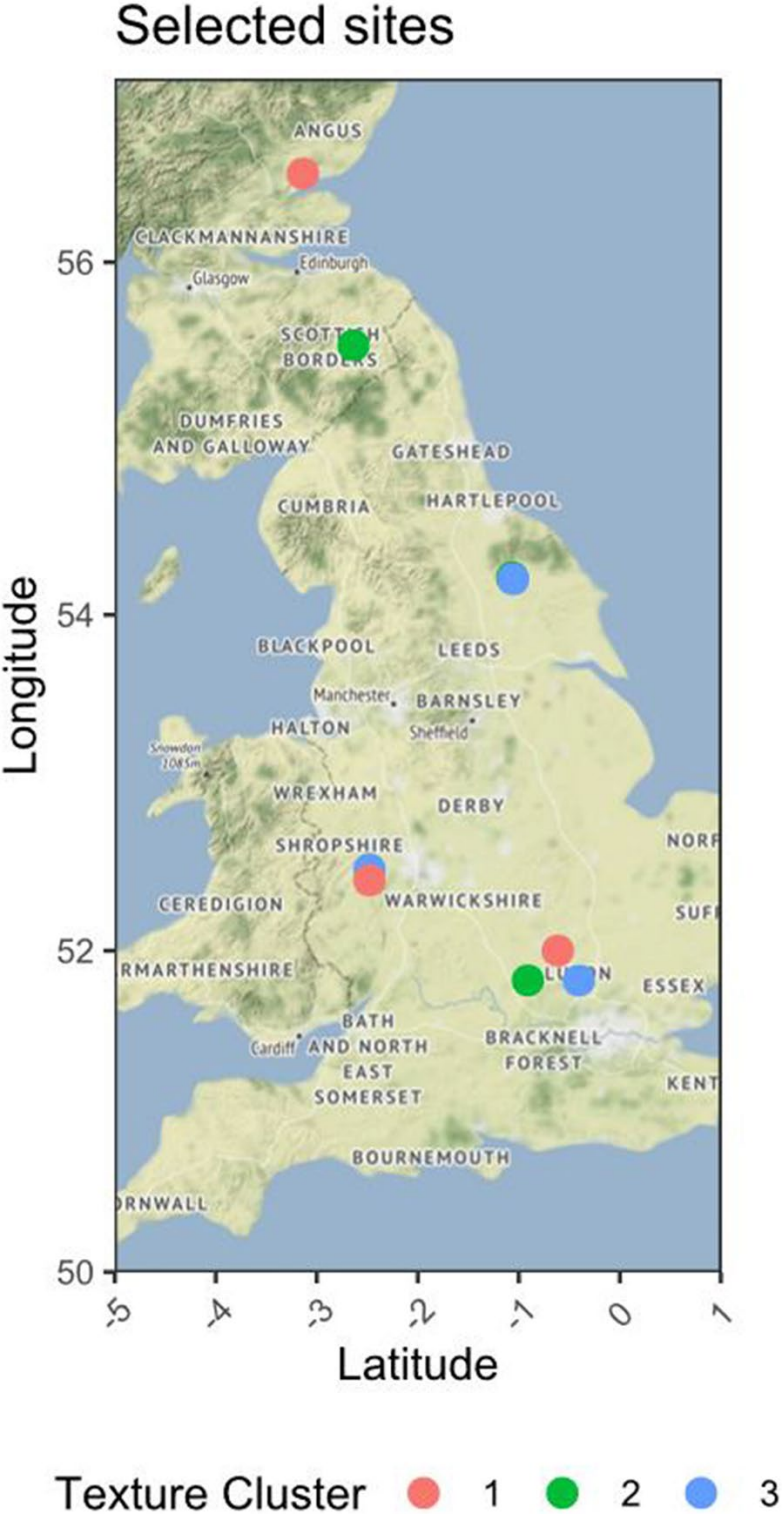
Soil sites from the BBSRC Assist Programme *JHI* and *Rres* sites. Soil texture data was clustered using complete linkage producing three major groups (Texture clusters 1, 2, and 3). Texture cluster 1 is formed by sandier soils (sandy loam-Red dot); cluster 2 is formed by clay soils (clay loam and clay = Green dot), while texture cluster 3 is formed by silt soils (clay loam and silt clay loam-Blue dot)

**Fig. 3:**
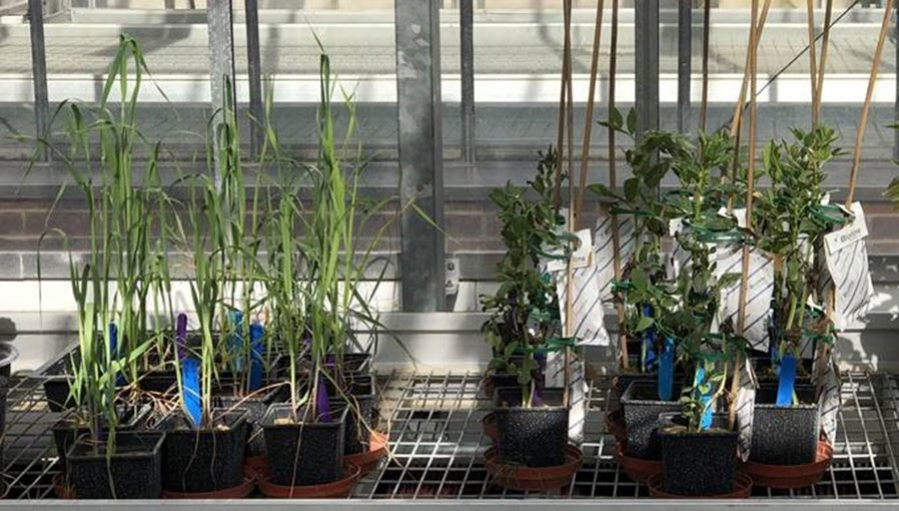
The Rothamsted pot experiment

The cryobank has applied a targeted approach to sample acquisition. Sample acquisition is integral to the success of the cryobank project (Fig. 1). At the outset of the project, it was decided that this would include soil and root material, culturable microorganisms and total community and microbial DNA. Six UK arable crops of global economic importance were targeted (wheat, barley, oats, oil seed rape, fava beans and sugar beet). Three contrasting soil types (silt, sandy loam and clay) were collected from 9 UK farm locations (3 of each soil, with pH of approximately 6.5) available through the Biotechnology and Biological Sciences Research Council (BBSRC) ASSIST programme network (Fig. 2) as well as other sites at Scotland’s Rural College (SRUC) and Rothamsted Research. Approximately 50 kg of soil was passed through a 5 mm sieve, then homogenised and used to culture plants without application of additional fertiliser from surface sterilised seeds in 20 cm pots under controlled glasshouse conditions for each crop, with 3 plants per pot. In total, five replicate pots for each of 6 crops (+ unplanted bulk soil) and 9 soil sites were prepared, equating to 315 pot systems on which the core resource is based (Fig. 3). Genomic DNA was prepared for culture-free descriptive assessments of all 315 samples. A strain library was prepared by culturing bacteria on 1/10th Tryptic Soy Agar (TSA) from the rhizosphere and bulk soil. Inoculant dilutions allowed up to 20–40 colony forming units per cultured plate, avoiding nutrient competition effects on isolate development. Additionally, fungi were isolated onto Tap Water Agar (TWA)/Malt Extract Agar (MEA) from a selection of samples using a standard dilution approach for a small subset of soils. This resulted in up to 96 microbial isolates per crop per pot system, and a unique collection of approximately 35,000 isolates was generated.

## Characterisation

All soils have been characterised including mineral analysis (N, P, K), and pH at Rothamsted, and the culture collection of 35,000 isolates is being screened for phenotypic traits. This includes the ability of microbial isolates to solubilise a range of macro and macro nutrients as well as abiotic stress tolerance. The community composition of the rhizosphere has been determined by ribosomal RNA gene amplicon sequencing for bacterial (16S) and fungal (ITS2) taxonomy. This allowed informed sample selection for higher resolution analysis using a shotgun metagenomic sequencing approach. Selection of samples for shotgun metagenomics sequencing reduces redundancy and contributes to cost efficiency. Metagenomics approaches, although not without limitations, are superseding lower-resolution amplicon sequencing for functional predictions, as technology develops (Xu and Zhao 2018). These datasets provide a comprehensive framework for downstream comparisons with microbiome analysis based on amplicon sequencing, and a snapshot of microbial communities using functional metagenome approaches. Selected culturable microbial isolates are currently being whole genome sequenced to provide understanding of the functional diversity as well as their phylogeny. This also permits, along with efforts in database harmonisation (McDonald et al. 2023), potential further integration of the different sequence-based datasets. Sequencing technology and data analysis approaches rapidly evolve, so any dataset is representative of a technological ‘snapshot’. Since the crux of the UKCMC is the generation of cryo-preserved samples, it future-proofs the resource for sample analyses that are not yet developed.

## Preservation and biobanking approaches

**Fig. 4:**
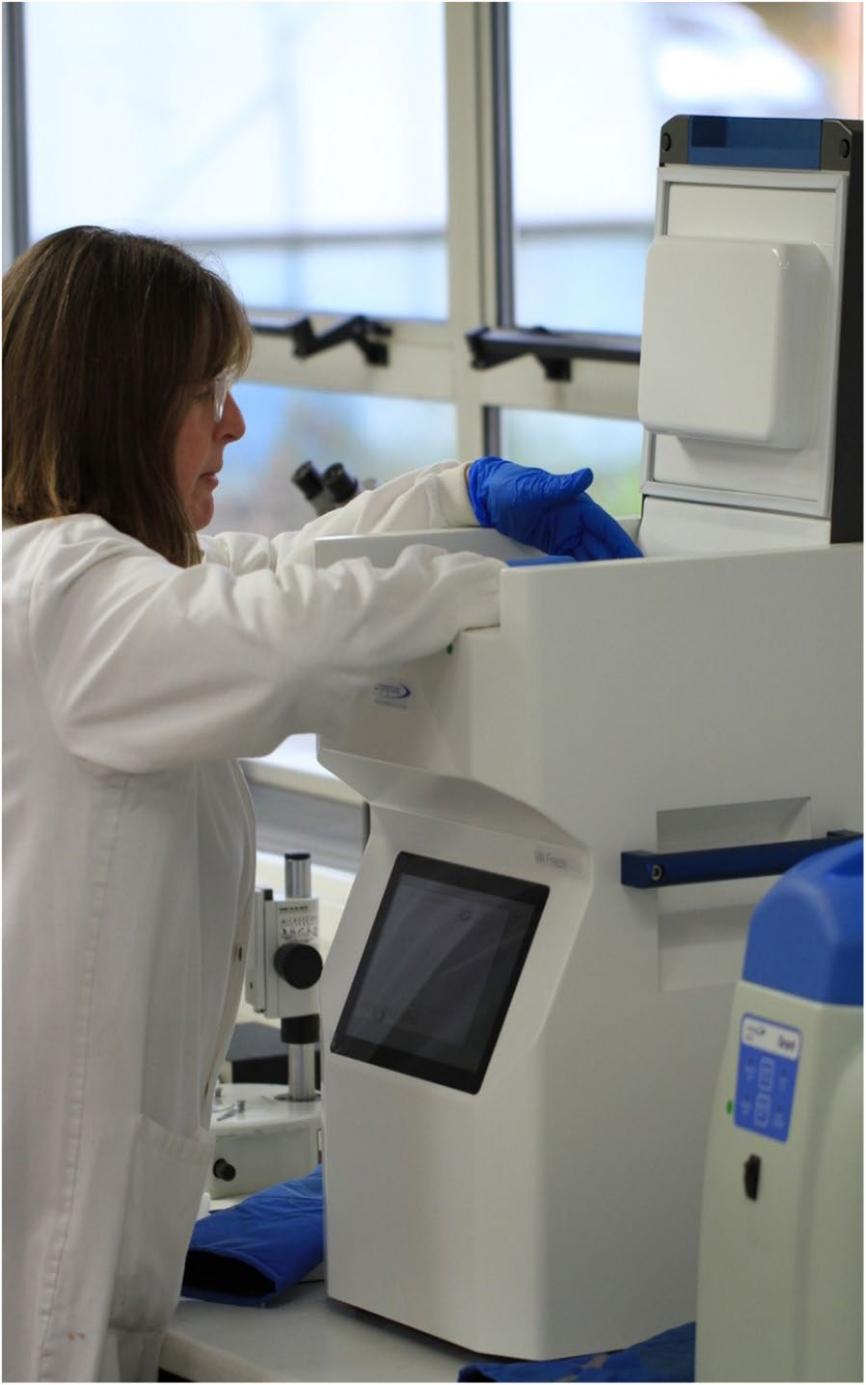
Via Freeze™ duo Stirling cycle cooler and operator

All samples generated are cryopreserved at ultra-low temperature in Statebourne Cryorefrigerators utilising liquid nitrogen as a refrigerant. This method was selected because of its high effectiveness and reliability (Ryan et al. 2019). Since the 1960s, culture collections have utilised storage at ultra-low temperature in a cryogenic gas or liquid as a method to preserve fungi and bacteria. Based on methodology developed by Polge et al. (1949) for spermatozoa, cryopreservation is regarded as the ’state of the art’ method for sample preservation. Even so, a careful approach is required to ensure sample integrity is not compromised during cooling, through the application of optimised techniques involving controlled rate cooling of samples (Ryan 2014) (Fig. 4). Furthermore, the preservation of recalcitrant strains often requires development of bespoke protocols such as encapsulation dehydration (Ryan 2001; Benson et al. 2018). This knowledge can be used to design protocols applicable to whole microbiome sample preservation. By combining two methodologies we can ensure that we capture more of the viable diversity within each microbiome sample. In this project we utilised controlled rate cooling using a ViaFreeze™ Duo Controlled rate cooler using a cooling regime of − 1 °C/min. Samples were cooled to − 80 °C before transfer to an automated Statebourne Biosystem 40 device with temperature held between − 175 and − 190 °C. Samples are not directly stored in liquid nitrogen but rather in a central chamber within the refrigerator. For the microbial isolates, we took a unique approach of preserving and storing them in 96 well microtiter plates. This ‘next generation, high throughput’ approach developed for use in biomedicine utilised a specially designed manifold plate holder for the Via Freeze cooler, where glycerol (10% w/v) was used as a cryoprotectant, and samples preserved using a cooling rate of − 1 °C/min. Plates are subsequently transferred to a Statebourne cryo-refrigerator and stored in stacks specifically designed for microtiter plates. A genomic assessment of the cryo-methodology has been undertaken that showed that controlled rate cooling does not adversely affect genomic integrity compared to control samples (Cafa et al. 2021). A further investigation that compared the functional profiles before and after cryopreservation demonstrated that functional potential is retained post cryopreservation (data not shown).

Identifiers for the project datasets (amplicon, metagenomic and in the future whole genome isolates) and associated metadata will be accessible through the project portal at AgMicrobiomeBase.org. The sequence data has been uploaded to public repositories (ENA and MGnify) and are interlinked through Biosamples entries. UKCMC is the first synchronised resource covering the soil microbiome of multiple crops in identical soil types, supported by resources integrated with public repositories. Uniquely, it maintains the link between soil health metrics, sample provenance, the physical sample and its metadata.

## Demonstrating the utility of the resource

The maintenance of the UK Crop Microbiome Cryobank ensures that this resource is available for academia as well as the Agritech sector to support the design of new biological products for sustainable agriculture. We are focussed on demonstrating the utility of the UKCMC for the isolation of PGPR and synthetic community construction. This will involve characterisation of the culturable microbiota associated with UK crop plants and the generation of crop-associated synthetic microbial communities (SynComs) with plant growth promoting traits. SynComs generated in this experiment will be added to the CryoBank and made available for other users and this can be used as a blueprint for use of the resource. Agricultural environments are known to profoundly shape microbial communities both in terms of overall genetic richness (Mehrabi et al. 2016) and the distribution and frequency of individual taxa (Mauchline et al. 2015; Pacheco-Moreno et al. 2021; Huang et al. 2019). These changes have been linked to specific root exudation profiles (Huang et al. 2019), root-associated differences in soil structure and the influence of farming practice and microbial biocontrol (Pacheco-Moreno et al. 2021). Changes in the distribution of key microbial species have in turn been linked to the development of disease suppressive soils and progressive changes in crop yields over time (Yang et al. 2011). Understanding the importance of crop-associated microbiomes for plant health is an important objective, both to build up a more complete picture of microbiome function and to demonstrate the utility and potential of the UKCMC for end-users.

## Facilitating research

The UKCMC will generate data to allow us to answer complex biological questions related to comparative, functional, and translational analysis. Furthermore, it will enable the research community to address questions such as:

i) How does plant growth promotion potential distribute within microbial phylogenies?
ii) Can we improve our understanding of population dynamics of microbial communities?
iii) How do microbiomes vary across soils and crops?
iv) Can we enable temporal analysis of microbiomes to measure changes caused by different agricultural practices or other abiotic factors?
v) Can we identify the microbial taxa and functional genes associated with soil and crop health?
vi) What other or additional microbial traits underpin soil and plant health?

## Conclusions: future sustainability and a model for other crops systems

Biobanking is expensive, and many critics consider the collecting and storage of microbes to be ‘stamp collecting’. However, as with Ryan et al. (2021), we support the argument that biobanks are crucial in-order-to underpin the stringency and reproducibility of microbiome research. In addition, they are a mechanism to preserve both agricultural and threatened biodiversity before the impact of human activities, climate change and predicted ecosystem collapse negatively impact the microbes present in our environment. The storage of microbial biodiversity is crucial in-order-to protect the environment and sustainable food production. The construction of the resource is funded under the UK’s BBSRC’s Bioinformatics and Biological Resources Fund. Its long-term viability relies upon utilisation of the resource by the research community. Provision of cultures and resources will be subject to a small charge to cover the cost of supply, with any excess revenues used to support the operation of the resource. Furthermore, the resource provides untapped biodiversity for industry to provide solutions to enhance sustainable agriculture. The ex-situ conserved repository also provides potential back-up in the event of a climatic or natural disaster, by providing resources to allow habitat reconstruction or microbes to enhance crop growth where the ‘in situ’ biodiversity may have become compromised. Governments and donor agencies, including trusts and foundations, should coordinate a funding approach to allow the UKCMC to be applied to the development of biological interventions for sustainable agriculture and for conservation efforts to mitigate human-led threats.

At the end of the research elements of the project, the legacy cryobank will be maintained alongside CABI’s culture collection, which has been in existence for over 75 years and provides a sustainability model for continuation. In parallel, the cryobank leaves a legacy of high-quality metadata to accompany the publicly available sequence data, strengthening its utility. The current UKCMC focuses on arable crops. However, the model is highly translatable to other crops in agri- and horticulture including global commodity crops such as tea, rice, cotton and coffee. Biobanking has a key future role to play, and without it, industry will not have the tools to develop new solutions, and the sustainability of our food systems will be at risk.
